# Supplementary material for: Resolving the full spectrum of human genome variation using Linked-Reads
Source: Genome Res. 2019 Apr;29(4):635–45. doi: 10.1101/gr.234443.118 (PMC6442396; doi:10.1101/gr.234443.118)
Supplement: Supplemental Material [file supp_gr.234443.118_Supplemental_longranger-2.2.zip › longranger-2.2.tar.bz2!/lr2.2-public/lib/stan/cmdstan/stan/doc/api/html/structstan_1_1gm_1_1error__codes.html]

Stan: stan::gm::error\_codes Struct Reference


|  |  |
| --- | --- |
|  | Stan  2.5.0  probability, sampling & optimization |


- Main Page
- Related Pages
- Namespaces
- Classes
- Files

- Class List
- Class Index
- Class Hierarchy
- Class Members

All Classes Namespaces Files Functions Variables Typedefs Enumerations Enumerator Friends Macros Pages

- stan
- gm
- error\_codes

Public Types |
List of all members

stan::gm::error\_codes Struct Reference

`#include <error_codes.hpp>`

|  |  |
| --- | --- |
| Public Types | |
| enum | {     OK = 0, USAGE = 64, DATAERR = 65, NOINPUT = 66,     SOFTWARE = 70, CONFIG = 78   } |
|  | |

## Detailed Description

Definition at line 7 of file error\_codes.hpp.

## Member Enumeration Documentation

anonymous enum

| Enumerator | |
| --- | --- |
| *OK* |  |
| *USAGE* |  |
| *DATAERR* |  |
| *NOINPUT* |  |
| *SOFTWARE* |  |
| *CONFIG* |  |

Definition at line 10 of file error\_codes.hpp.

---

The documentation for this struct was generated from the following file:

- src/stan/gm/error\_codes.hpp


---

[ Stan Home Page ]

*© 2011–2014,
Stan Development Team.*
